# Cardiomyocyte Proliferation as a Source of New Myocyte Development in the Adult Heart

**DOI:** 10.3390/ijms22157764

**Published:** 2021-07-21

**Authors:** Jaslyn Johnson, Sadia Mohsin, Steven R. Houser

**Affiliations:** Independence Blue Cross Cardiovascular Research Center, Lewis Katz School of Medicine, Temple University, Philadelphia, PA 19140, USA; jaslyn.johnson@temple.edu (J.J.); sadia.mohsin@temple.edu (S.M.)

**Keywords:** cardiomyocyte proliferation, cardiac regeneration, cardiomyocyte cytokinesis, myocardial infarction

## Abstract

Cardiac diseases such as myocardial infarction (MI) can lead to adverse remodeling and impaired contractility of the heart due to widespread cardiomyocyte death in the damaged area. Current therapies focus on improving heart contractility and minimizing fibrosis with modest cardiac regeneration, but MI patients can still progress to heart failure (HF). There is a dire need for clinical therapies that can replace the lost myocardium, specifically by the induction of new myocyte formation from pre-existing cardiomyocytes. Many studies have shown terminally differentiated myocytes can re-enter the cell cycle and divide through manipulations of the cardiomyocyte cell cycle, signaling pathways, endogenous genes, and environmental factors. However, these approaches result in minimal myocyte renewal or cardiomegaly due to hyperactivation of cardiomyocyte proliferation. Finding the optimal treatment that will replenish cardiomyocyte numbers without causing tumorigenesis is a major challenge in the field. Another controversy is the inability to clearly define cardiomyocyte division versus myocyte DNA synthesis due to limited methods. In this review, we discuss several studies that induced cardiomyocyte cell cycle re-entry after cardiac injury, highlight whether cardiomyocytes completed cytokinesis, and address both limitations and methodological advances made to identify new myocyte formation.

## 1. Introduction

Cardiovascular diseases such as myocardial infarction (MI) are a major cause of human mortality [1]. MI is caused by occlusion in the coronary arteries which deprives downstream cardiac tissue of normal blood and oxygen supply, resulting in the death of cardiomyocytes. Then, the heart undergoes pathological remodeling, including inflammation, scar formation, left ventricular (LV) dilation, LV wall thinning, and decreased pump function [2,3,4]. Activation of neurohormonal systems including the sympathetic nervous system partially maintains cardiac output, at least at rest. However, persistent neurohormonal signaling and increased wall stress leads to hypertrophy of surviving cardiomyocytes, dilation of the left ventricle, and deterioration of myocardial contractility and eventually these changes can progress to heart failure (HF) or death [5,6]. Current therapies for MI work to quickly reopen the blocked artery and restore blood flow to the ischemic heart. This rescues some of the viable cardiomyocytes in the affected tissue; however, re-establishing blood flow causes ischemia-reperfusion injury and further damage to cardiac myocytes in the infarct core [7]. Inevitably, the heart still undergoes adverse cardiac remodeling with reduced pump function. Regenerative therapies that can replace lost cardiomyocytes, and thereby restore cardiac contractility would be the optimal therapy for MI patients, because this would address the actual cause of the heart failure.

Studies in lower vertebrates such as zebrafish and newts have demonstrated that their hearts can regenerate after injury by proliferation of pre-existing cardiomyocytes [8]. The adult mammalian heart is thought to be a post-mitotic organ; however, it is well known that immediately after birth, normal mammals generate large numbers of new cardiomyocytes until about postnatal day 7, when new myocyte formation slows to a very low rate [9,10]. The rate of new myocyte formation appears to increase about four-fold following MI injury, with pre-existing cardiomyocytes near the infarct area being the dominant source of new myocytes [11]. The rate of new myocyte formation is still very low and insufficient to replace the myocytes killed by the MI. It appears that human cardiomyocytes can renew throughout life with a starting rate of about 1% turnover per year, which declines to 0.3% by 75 years of age [12,13]. The process of cardiomyocyte regeneration requires cardiac myocytes to undergo dedifferentiation during cell cycle re-entry [14,15], completion of cell division, and then redifferentiation into mature cardiac myocytes [16]. Many studies have explored approaches to manipulate the cellular mechanisms involved in the cardiomyocyte cell cycle to enhance myocyte regeneration. Understanding if/how these mechanisms can be altered to induce a more robust regeneration of cardiomyocytes could lead to therapies to replenish cardiomyocyte numbers and restore cardiac pump function after injury.

## 2. Main Text

### 2.1. Targeting Cell Cycle Regulators to Induce Cardiomyocyte Proliferation

**Figure 1:**
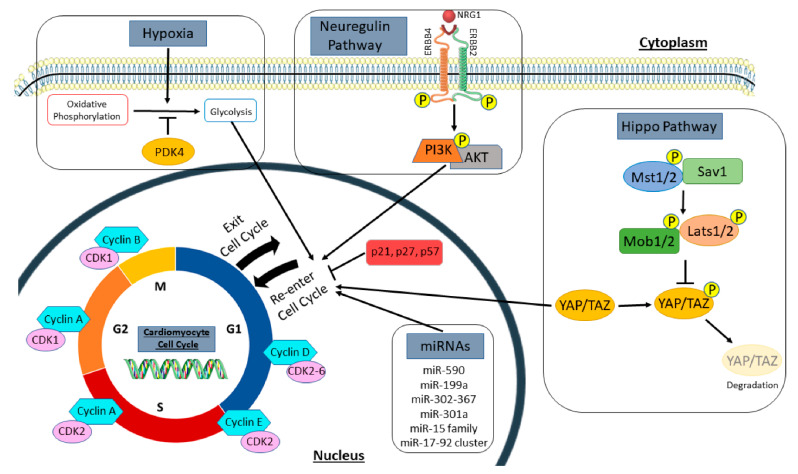
Regulators of the cardiomyocyte cell cycle. Summary of signaling pathways, cell cycle genes, and extracellular stimuli that induce cardiomyocyte proliferation, as discussed in this review. This includes cell cycle promotors and inhibitors, both the hippo and neuregulin pathways, microRNAs, and metabolic regulators, such as hypoxia and PDK4. Cyclins and cyclin-dependent kinases promote cell cycle activation and progression, while cyclin-dependent kinase inhibitors such as p21, p27, and p57 inhibit the cell cycle. In the hippo pathway, activation of upstream kinases (Mst1/2, Sav1, Mob1/2, and Lats1/2) phosphorylate the downstream transcription co-activators (YAP/TAZ) to promote their degradation. However, inactivation of upstream kinases causes YAP/TAZ to translocate to the nucleus to promote cell cycle re-entry in cardiomyocytes. Neuregulin (NRG1) binds to its tyrosine receptor subunits (ERBB2 and ERBB4) to activate a downstream signaling pathway (PI3K/AKT) and induce myocyte proliferation. Several microRNAs (miRNAs) also regulate the cell cycle to influence new myocyte formation. Hypoxia and PDK4 lead to a metabolic switch in cardiomyocytes from mitochondrial oxidative phosphorylation to anaerobic glycolysis for energy production. This metabolic change influences cell cycle activation in cardiomyocytes. PDK4, pyruvate dehydrogenase kinase 4.

Cardiomyocytes exit the cell cycle shortly after birth and are thought to be terminally differentiated [17]. However, there is evidence that mature cardiomyocytes can re-enter the cell cycle but at a lower rate than during the neonatal time period [10,12]. The post-mitotic cardiac myocytes decline in proliferative capacity is thought to be due to a downregulation of cell cycle promoters and upregulation of cell cycle inhibitors [18]. Therefore, many studies have focused on identifying the mechanisms of cell cycle reactivation in cardiomyocytes (Figure 1), as a potential therapeutic target for cardiac regeneration (Table 1). Cyclins and cyclin-dependent kinases (CDKs) are cell cycle regulators known to promote activation and progression through the cell cycle. Transgenic overexpression of cyclin D1 in murine cardiomyocytes was found to activate DNA synthesis in >40% of adult cardiac myocytes that was sustained over time; however, it was shown that cycling cardiomyocytes underwent endoreplication, where they increased their nuclear gene content, without completing karyokinesis or cytokinesis [19,20]. Overexpression of cyclin D2 in post-MI hearts induced cardiomyocytes to re-enter the cell cycle and undergo division as evidenced by the colocalization of X-gal and histone H3, markers for DNA synthesis and mitosis, respectively. Expression of cyclin D2 promoted DNA synthesis at 7 days post MI and persisted over time with increased cardiac mass and reduced infarct size at 5 months post MI [21]. Similar results were seen in a transthoracic aortic constriction (TAC) mouse model where cyclin D2 enhanced cardiomyocyte proliferation, reduced fibrosis, and prevented cardiac dysfunction [22]. Cyclin A2 has also been well studied; mice have been shown to constitutively expressing cyclin A2 in the heart develop cardiac hyperplasia due to the heightened rate of cardiomyocyte proliferation observed from postnatal day 8 up to 1.5 years of age [23]. Nevertheless, these mice were protected from developing heart failure post MI, as they exhibited improved cardiac function and increased mitosis of cardiomyocytes in the infarct and border zones of the injured heart [24]. Other studies have delivered cyclin A2 to the heart by injection of an adenoviral vector in a heart failure rat model or by injection of cyclin A2-encoding adenovirus into an MI pig model. In both cases, cyclin A2 promoted cardiac myocytes to re-enter the cell cycle and complete cytokinesis, which led to improved pump function of the ischemic heart [25,26]. Additionally, other cell cycle regulators such as cyclin B1 have been shown to improve the proliferative capacity of neonatal and adult rat cardiomyocytes [27], and overexpression of CDK2 in the heart elevated cardiomyocyte DNA synthesis and increased the number of small mononucleated cardiomyocytes in the heart [28].

Inhibition of cyclin-dependent kinase inhibitors (CKIs) has also been shown to reactivate the cell cycle in cardiac myocytes. Triple knockdown of CKIs including p21, p27, and p57 induced myocyte proliferation with evidence of karyokinesis and cytokinesis in neonatal and adult cardiomyocytes [29]. Likewise, inactivation of cyclin-dependent kinase inhibitor 2a (CDKN2a) or downregulation of p21 alone induced cardiomyocytes to enter mitosis and synthesize DNA [30,31]. Numerous studies on regulating various cell cycle transcription factors including E2F-1, E2F2, Meis1, Tbx6, and Tbx20, have also provided evidence that the proliferative potential of adult cardiac myocytes can be enhanced to promote new myocyte formation from pre-existing cardiomyocytes [32,33,34,35,36,37]. Recently, a combination treatment of cell cycle activators and inhibitors induced 15–20% of cardiomyocytes to re-enter the cell cycle and divide, reduced scar size post MI, and attenuated cardiac dysfunction [38]. Although these studies prove that cardiomyocyte proliferation can be achieved to promote cardiac regeneration and repair in cardiovascular diseases, there is still a limitation in the number of new myocytes formed. In many of these findings, cardiomyocyte cell cycle activity was re-activated and promoted DNA synthesis but resulted in multinucleation or polyploidy without complete cytokinesis. Further study of signaling pathways in the cardiomyocyte cell cycle is needed to determine how to induce cardiomyocytes to enter the cell cycle and divide to form two daughter cells.

### 2.2. Manipulation of Signaling Pathways to Promote New Myocyte Formation

Embryonic heart development and postnatal myocyte maturation are regulated by multiple signaling pathways that have been modulated to stimulate cardiomyocyte proliferation. One of the conserved signaling cascades involved in myocyte growth is the hippo pathway. This pathway functions to restrict cardiomyocyte proliferation through phosphorylation-mediated inactivation of its downstream transcription co-activators YAP/TAZ, to cause cardiomyocytes to withdraw from the cell cycle. Therefore, inactivation of hippo signaling in embryos results in enlarged hearts from overactivation of the cardiomyocyte cell cycle [53]; conversely, embryonic deletion of YAP caused fetal death due to cardiac hypoplasia, whereas a constitutively active form of Yap resulted in upregulation of β-catenin, a known inducer of cardiomyocyte proliferation, and increased myocardial mass through cardiomyocyte proliferation rather than hypertrophy [54,55]. The interaction of YAP and TEAD transcription factors in the hippo pathway is required for cardiomyocytes to re-enter the cell cycle and divide [55], as conditional knockout of TEAD1 in murine neonates lead to complete lethality by postnatal day 9 [56]. Understanding the role of the hippo pathway in cardiac development has led to investigations that explored the idea that the hippo signaling cascade can be altered to promote cardiac regeneration following injury. These studies have shown that overexpression of YAP in cardiomyocytes attenuated myocardial fibrosis, increased cardiomyocyte cell cycle activity, and improved cardiac function following MI in neonatal and adult mice [57]. Likewise, AAV9 delivery of human YAP in adult mice hearts induced cardiomyocyte mitosis, improved cardiac contractility, and enhanced mice survival after MI [39]. In contrast, heterozygous deletion of YAP increased cardiomyocyte apoptosis and prevented myocyte hypertrophy after MI, which led to deleterious cardiac remodeling and poor pump function [58].

Other hippo-related genes can also induce adult cardiomyocytes to re-enter the cell cycle. Inactivation of the hippo pathway by conditional knockout of its regulatory Salv gene in adult mice has been shown to reduce scar size, induce new myocyte formation, and attenuate cardiac dysfunction in MI and HF disease models [40,41]. Related studies have shown that adult myocyte overexpression of YAP alters their gene expression to resemble embryonic myocytes in the cell cycle. More specifically, YAP reorganizes chromatin accessibility in TEAD and AP1 motifs to activate transcription of fetal and proliferative genes in cardiomyocytes [59]. YAP also binds to the B-MYB subunit of the Myb-MuvB (MMB) complex, which regulates mitotic genes, to activate transcription of cell cycle genes in cardiomyocytes and promote their proliferation [60].

The neuregulin/ERBB2/ERBB4 pathway is also involved in cardiomyocyte proliferation. Neuregulin (NRG1) is an extracellular mitogen that binds to its tyrosine receptor subunits ERBB2 and ERBB4 to activate downstream signaling pathways including the PI3K/AKT pathway to induce cardiomyogenesis during fetal heart development. In adult mammals, the neuregulin pathway can activate the cell cycle in mature cardiomyocytes to induce proliferation. Treatment of adult rat ventricular myocytes with NRG1 promoted mostly mononucleated cardiomyocytes to re-enter the cell cycle and complete cytokinesis [42]. In vivo, overexpression of ERBB4 in the postnatal heart induced DNA synthesis in myocytes, and injection of recombinant NRG1 in control or post-MI adult mice stimulated new myocyte formation by cardiomyocyte proliferation and not from progenitor cell differentiation [42]. Similarly, overexpression of ERBB2 in all ages of mice caused cardiac enlargement due to widespread cardiomyocyte proliferation and hypertrophy; however, transient induction of ERBB2 in adult hearts post MI induced cardiomyocyte dedifferentiation, proliferation, and redifferentiation to promote a more limited cardiac regeneration that improved heart function [43]. Activations of the NRG1/ERBB2 signaling caused a metabolic switch in border zone cardiac myocytes from oxidative phosphorylation to glycolysis, with an upregulation in glycolysis genes and a reduction in mitochondrial function [61]. This metabolic reprogramming induced adult murine cardiomyocytes to re-enter the cell cycle and divide, resembling embryonic cardiac myocytes [61].

Other signaling pathways, including PI3K/AKT/CDK7, Wnt/β-catenin, PDGFR-β, and Notch signaling pathways can also stimulate cardiomyocytes to proliferate and induce cardiac regeneration during heart disease or injury [62,63,64,65]. Furthermore, several studies have discovered that multiple mitotic pathways within cardiomyocytes can crosstalk to stimulate proliferation of pre-existing cardiomyocytes, thus, increasing myocardial mass and promoting cardiac repair [54,66,67,68]. One study showed that transient overexpression of ERBB2 activated YAP mechanotransduction signaling and an epithelial-mesenchymal transition-like response in cardiomyocytes that induced cardiac regeneration in a HF murine model [69]. Collectively these studies show that manipulation of several cell cycle pathways, including hippo and neuregulin signaling, can induce cardiomyocyte mitosis and attenuate cardiomyocyte necrosis and cardiac dysfunction following injury. A caution is that prolonged modulation of these pathways can lead to hypertrophic cardiomyopathy [43] or heart failure, where persistent inactivation of the hippo pathway causes extensive cardiomyocyte dedifferentiation which impairs pump function and repair of the MI injured heart [70]. Therefore, controlled, transient regulation of these pathways needs to be developed as a strategy to replace myocytes lost to disease, and thereby improve myocardial function following injury.

### 2.3. Selective Induction/Inhibition of Gene Expression can Induce Cell Cycle Re-Activation in Cardiac Myocytes

MicroRNAs (miRNAs) regulate post-transcriptional gene expression and several miRNAs have been linked to cardiomyocyte proliferation. High throughput functional screening has identified 40 human miRNAs that promote cardiomyocyte growth and division, with miR-590 and miR-199a as the major regulators of this process in neonatal and adult rodents [71]. Following MI, therapeutic delivery of both miRNAs with an AAV9 vector improved cardiac contractility, reduced scar size, and increased cardiomyocyte proliferation [71]. Similar results were reported when a single intracardiac injection of synthetic human miR-590-3p and miR-199a-3p mimics promoted cardiac regeneration and increased cardiac function in mice post MI [44]. Expression of miR-199a also benefited ischemic hearts of a large animal model [45]. Another mitogen that was initially known as an oncogene, is the *miR-17-92* cluster, which is required for cardiomyocyte proliferation in embryonic and neonatal hearts; overexpression of this cluster in the heart can induce cardiomyocyte DNA synthesis at all ages in mice and following cardiac injury, thus, protecting the heart from MI-induced remodeling and dysfunction [46]. Of this cluster, miR-19a/19b are most potent at stimulating cardiac regeneration by causing enhanced cardiomyocyte proliferation, inhibition of apoptosis and proinflammatory immune responses, reduced fibrosis, and improved pump function up to 12 months post MI [47]. Alternatively, the miR-15 family plays a role in cardiomyocyte withdrawal from the cell cycle and is upregulated in cardiac ventricles by postnatal day 10, with miR-195 being the most elevated [72]; miR-195 represses several cell cycle genes including checkpoint kinase 1 (*Chek1*) to inhibit myocyte proliferation after birth. Thus, knockdown of the miR-15 family increased mitosis in neonatal cardiac myocytes [72] and in adult mice which was protective against MI-induced cardiac injury [48].

Several miRNAs have also been shown to regulate genes in signaling pathways that influence cardiomyocyte mitosis. The miR-302-367 cluster represses signal transduction in the hippo pathway to induce cardiomyocyte proliferation and this cluster is required for embryonic development, as cardiac knockdown of miR-302-367 results in hypoplasia [49]. Therapeutically, transient expression of miR-302b/c, a member of the miR-302-367 cluster, increased cardiac mass and enhanced heart function in an MI mouse model [49]. Likewise, other pro-proliferative miRNAs, including miR-199a-3p, activate YAP in the hippo pathway to enhance DNA synthesis in cardiomyocytes. More specifically, miR-199a-3p downregulates YAP inhibitory kinase, TAOK1, and the E3 ubiquitin ligase, β-TrCP, to inhibit YAP degradation, and also represses Cofilin2 to maintain YAP nuclear localization [73]. PTEN/PI3K/AKT is another signal transduction pathway that is activated by miR-301a to promote cardiomyocyte re-entry into the cell cycle; miR-301a suppresses PTEN to activate AKT and induce expression of cell cycle genes such as cyclin D1, in cardiac myocytes [50]. Other miRNAs have been reported to promote cell cycle progression in cardiomyocytes by targeting the TBX1/JAK2/STAT1 pathway [74], the NFkB pathway [75], or signaling molecules such as the cell cycle inhibitors Wee1 [76] and Bim [77], among others [78,79]. These studies suggest that MiRNAs possess the potential for replenishing cardiomyocyte numbers in injured hearts. A cautionary note is that prolonged expression of miRNAs can lead to cardiac arrhythmias and death from an accumulation of dedifferentiated and hyper-proliferative cardiac myocytes [45,49]. Therefore, it appears that controlled, transient expression of miRNAs is an essential component in the use of these molecules to prevent/repair cardiac damage [49].

### 2.4. Changes in Cardiac Myocyte Metabolism Can Induce Cell Cycle Re-Entry

During embryonic development, the mammalian heart primarily utilizes anaerobic metabolism to produce energy and fetal cardiomyocytes have a high proliferative capacity; immediately after birth, the heart is exposed to blood with a much greater oxygen content and there is a switch to aerobic metabolism to fuel the increased contractile and functional requirements. This switch to higher oxygen levels is associated with the exit of cardiomyocytes from the cell cycle and reduces their mitotic potential [80]. Neonatal exposure to hyperoxia resulted in the production of reactive oxygen species (ROS), oxidative DNA damage, and activation of the DNA damage response (DDR) pathway which all reduce cardiomyocyte proliferation. Hypoxia treatment of neonates maintains postnatal proliferation of cardiac myocytes and inhibits oxidative stress [80]. These finding suggest that fetal cardiomyocytes require hypoxia for cell cycle progression during cardiac development, which is mediated by HIF1α expression in embryonic hearts [81]. Adult hearts may contain a small group of hypoxic cardiomyocytes that are mononucleated and can re-enter the cell cycle and divide [82]. The low cardiomyocyte renewal rate (<1% each year) in healthy adults [12] could be the result of the higher oxygen content of blood in adult versus fetal hearts. The increased number of “new” cardiac myocytes after ischemic injury could be the result of exposure of adult myocytes to a hypoxic environment [51].

**Figure 2:**
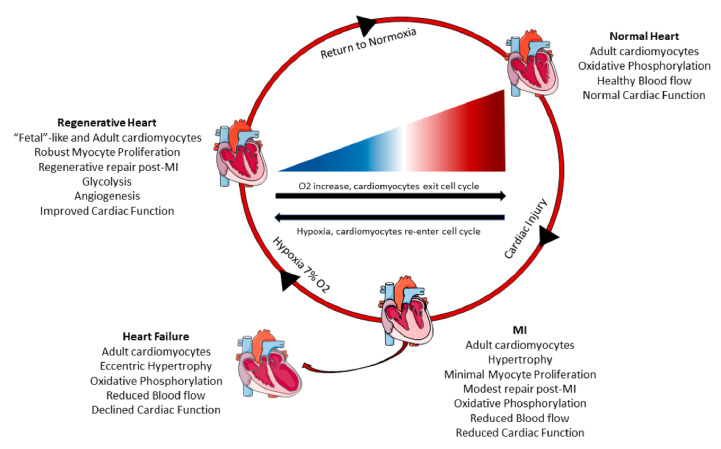
Systemic hypoxia can induce cardiomyocyte proliferation and cardiac regeneration. Healthy adult hearts have normal cardiac function, blood flow, and utilize oxidative phosphorylation to generate energy. Following myocardial infarction (MI), there is increased cardiomyocyte hypertrophy, scar formation, reduced cardiac pump function, and minimal cardiomyocyte proliferation. These adverse changes can progress and lead to heart failure overtime with increased dilation of the myocardium, poor blood circulation, and declined cardiac function. However, a recent study in mice [51] demonstrated that systemic hypoxemia post MI caused cardiomyocytes to undergo a metabolic switch from oxidative phosphorylation to anaerobic glycolysis. Hypoxia also enhanced cardiomyocyte proliferation and hypoxic myocytes resembled fetal cardiomyocytes. Hypoxic mice had increased angiogenesis, reduced scar size, and improved cardiac function. When the mice returned to normoxia, cardiac repair and elevated cardiac pump function was sustained.

Previous studies have shown hypoxic preconditioning of cardiac myocytes limited ROS production, decreased cardiomyocyte death, and reduced infarct size following prolonged ischemia in rodents and large mammals [83,84,85]. In patients with acute ST segment elevation myocardial infarction (STEMI), remote upper limb ischemic preconditioning followed by percutaneous coronary intervention exhibited improved ejection fraction and reduced HF and cardiac death outcomes over a 12-month follow-up [86]. Remote ischemic preconditioning was shown to provide cardioprotection and attenuate ischemia-reperfusion injury by enhancing anaerobic glycolysis in patients undergoing coronary artery surgery [87]. Thus, pre-exposure of the myocardium to hypoxia can decrease cardiac damage, salvage cardiomyocytes in the infarct region, and reduce oxidative stress in adult hearts, mimicking neonatal outcomes under hypoxia [80]. More specifically, a recent study investigated the effects of hypoxemia on myocyte proliferation in adult mammals. Gradual reduction of ambient oxygen levels to 7% oxygen induced a proliferative response in cardiac myocytes [51]. Hypoxemia in mice reduced mitochondrial oxidative metabolism and ROS production, repressed oxidative DNA damage, and caused cardiac hyperplasia due to increased cardiomyocyte mitosis [51]. Following MI, hypoxia treatment improved cardiomyocyte renewal, decreased fibrotic area, and increased cardiac function [51]. Similar results have been seen in cardiac tissue specimens from postnatal human patients with tetralogy of Fallot, where moderate hypoxia reduced oxidative DNA damage and increased cardiomyocyte proliferation [88]. These studies suggest that hypoxia induced pre-existing cardiomyocytes to re-enter the cell cycle and divide by altering the mitochondrial metabolism (Figure 2).

Fetal mitotic cardiomyocytes utilize pyruvates in anaerobic glycolysis to generate energy, but by postnatal day 7, they switch to utilize fatty acids in mitochondrial oxidative phosphorylation for energy and most cardiomyocytes have exited the cell cycle. Reversal of this process can prolong cardiomyocyte proliferation, as neonatal mice fed a fatty acid deficient diet extended the proliferative window of cardiomyocytes up to 3 weeks postnatally [52]. In adults following MI, therapeutic deletion of pyruvate dehydrogenase kinase 4 (PDK4) increased glucose oxidation which induced cardiomyocyte mitosis, and attenuated adverse cardiac structure and function. Likewise, pharmacological inhibition of PDK4 promoted cardiomyocyte proliferation with complete cytokinesis [52]. Together, alteration in mitochondrial metabolism to reduce fatty acid utilization can induce cell cycle re-entry in cardiomyocytes. Confirmation of these findings would enhance confidence for translating this novel therapy into large animal models and patients. Interestingly, humans living at higher altitudes have reduced mortality from coronary heart disease [89,90,91], suggesting that hypoxemia and modulation of mitochondrial oxidative metabolism may offset those factors that induce cardiac diseases.

### 2.5. Limitations in Cardiomyocyte Proliferation Techniques

Several methods have been employed to study cardiomyocyte proliferation (Table 2). Most commonly, cardiomyocyte cell cycle activity is measured by 5-ethynyl-2′-deoxyuridine (EdU) incorporation into new DNA or the presence of proteins only expressed during the cell cycle, such as Ki67, phosphorylated histone H3 (pHH3), or Aurora B. EdU is incorporated into newly formed DNA, Ki67 is expressed in the G1 to M phase of the cell cycle, pHH3 is expressed during chromosome condensation, and Aurora B is expressed during mitosis and during cell division [92]. Many studies have concluded that new myocytes are present (with cytokinesis) when these cell cycle markers are found. However, other possibilities include endoreplication, endomitosis, and DNA damage/repair. Most of these markers indicate cell cycle re-entry and DNA synthesis, but they do not reliably determine if cardiomyocytes undergo cell division, polyploidy, or polynucleation. Therefore, these represent markers of cell cycle activity that may or may not lead to cell division and the production of new cardiomyocytes.

A number of mouse models have been generated to provide more direct evidence of cardiomyocyte renewal. Mosaic analysis with double markers (MADM) mouse models fluorescently label cardiomyocytes that have completed cytokinesis. Using Cre-loxP recombination, daughter cells are single-labeled green or red after cell division [93]. This model was used to validate low cardiomyocyte turnover in adults and that new myocytes formed after birth or post MI are derived from pre-existing cardiomyocytes [9]. Rainbow, also referred to as Brainbow mice, tracks clonal expansion of cardiomyocytes. Following Cre-mediated recombination, several myocytes are randomly labeled with one of four fluorescent colors and analysis of the hearts at terminal studies confirms proliferation by single-color clusters, indicating one myocyte divided to generate adjacent myocytes. This mouse model was used in a lineage tracing study on clonal distribution during cardiac development [94], and corroborated increased cardiomyocyte proliferation when YAP was overexpressed in the heart [39]. Another mouse model where cardiomyocytes express fluorescence ubiquitination-based cell cycle indicators (FUCCI) specifically revealed when cells progress from G1 to end stages of mitosis in the cell cycle. Cardiac myocytes fluorescently express monomeric Kusabira Orange (mKO) when they are in the G1 phase and express Azami Green (AzG) when they are in S, G2, or M phases of the cell cycle; dual labeling of both colors indicates cell cycle arrest [95]. This allows a temporal view of cardiomyocyte cycling through the cell cycle with cytokinesis identified by the presence of two colorless daughter cells.

More recently, an Aurora Kinase B (Aurkb) mouse system was generated to label cardiomyocytes red when Aurora B was expressed during mitosis and cytokinesis [96]. This approach tracks myocytes that undergo cell proliferation and division, which eliminates controversial problems of Aurora B staining including non-specific staining and transient expression during mitosis. Other new methods to detect cardiomyocyte proliferation involves using molecular beacons to target anaphase and telophase/cytokinesis mRNA in cardiomyocytes followed by cell sorting to identify cycling myocytes that complete cell division [97]. This technique allows isolation of a purer population of dividing cardiomyocytes by eliminating myocytes that only undergo endoreplication or endomitosis; however, this technique is limited to in vitro and ex vivo experiments. In addition, quantification of cardiomyocytes by stereological practices provides measurements of cardiomyocyte number and nucleation, which can determine the amount of new myocyte formation as compared with control hearts [98]. These new systems should be used to confirm whether previously reported data on cardiomyocyte renewal actually represents the generation of new cardiac myocytes by accurately identifying cell cycle progression and completion of mitosis in cardiac myocytes.

## 3. Conclusions

The human heart is believed to be a post mitotic organ, but numerous studies in animals and humans have provided evidence suggesting there can be a low rate of myocyte renewal. The mammalian heart has a high myocyte proliferative capacity following birth, but after several days, these cells exit the cell cycle. There are only a few cardiomyocytes that maintain their proliferative potential throughout life which is likely responsible for the yearly 1% cardiomyocyte renewal in humans. Following cardiac injury, embryonic and postnatal animals can completely or partially regenerate their myocardium by proliferation of pre-existing cardiomyocytes. In adults, few cardiomyocytes re-enter the cell cycle and divide after injury, which leads to adverse cardiac remodeling and function. Studies of embryonic cardiac development and postnatal cardiomyocytes have identified regulators of the cardiomyocyte cell cycle that can be manipulated in adults to induce cardiomyocyte mitosis.

Overexpression of cell cycle promoters such as cyclins and CDKs enhance cardiomyocyte proliferation and prolonged expression can result in cardiac hyperplasia. These cell cycle stimulators are reparative in MI and TAC injury models by promoting new myocyte formation, reducing fibrosis, and attenuating cardiac pump malfunction. Likewise, repression of CKIs or cell cycle inhibitors induce DNA synthesis in cardiac myocytes which can be synergistically enhanced when combined with cell cycle activators. Signaling pathways involved in cell cycle progression such as the neuregulin/ERBB2/ERBB4 pathway or in cardiomyocyte cell cycle arrest including the hippo-YAP pathway have been targeted to promote proliferation of pre-existing myocytes. Upregulation of YAP or inhibition of hippo genes in neonatal and adult hearts increase cardiac mass by inducing proliferation of mature cardiac myocytes. In disease models, modulation of this pathway improves cardiac repair and animal survival, but persistent activation of YAP can leave many cardiomyocytes in a dedifferentiated state which can lead to heart failure. However, regulation of YAP by transient expression of miR-302-367 has been reported to prevent heart failure and increased cardiac regeneration following MI. Other miRNAs have also been reported to work independently or in conjunction with several signaling pathways to reactivate the cardiomyocyte cell cycle and promote division, thus, improving MI outcomes. Importantly, miRNA modulation of mitotic gene expression and manipulation of cell cycle signaling pathways need to be tightly controlled to restore cardiomyocyte numbers after injury without provoking tumorigenesis or accumulation of immature cardiac myocytes. Recent studies have suggested that hypoxemia in adult mice could induce proliferation of cardiomyocytes in healthy and post MI hearts by causing a metabolic switch in cardiomyocyte mitochondrial oxidation, from oxidative phosphorylation to glycolysis. These studies only showed modest improvement in cardiac function and cardiomyocyte proliferation, and while myocytes may have entered the cell cycle and increased DNA synthesis, it was not clear that they completed cytokinesis and generated new myocytes. More research is required to discover new pathways and mechanisms that can influence cardiomyocyte division and avoid endoreplication or endomitosis. Implementation of these practices can increase the therapeutic potential of these treatments for enhancing cardiac regeneration and repair following injury.

An obstacle in determination of new myocyte formation is the accurate interpretation of studies using proliferative markers such as EdU, Ki67, pHH3, and Aurora B. Most of these markers indicate cell cycle re-entry and DNA synthesis but only Aurora B is present during cytokinesis [99]. However, Aurora B antibodies can have nonspecific staining and lead to false conclusions [92]. Several new transgenic mouse models have been established to define cardiomyocyte proliferation more clearly. MADM mice label daughter cells of cardiac myocytes that have successfully completed cytokinesis, while Rainbow mice analyze clonal expansion of a single-colored myocyte that divided to form a cluster of same-colored myocytes. Aurora kinase B mice also label dividing cardiac myocytes. Therefore, these three mouse models are available to define cardiomyocyte division with complete cytokinesis. FUCCI mice are better suited for in vitro analysis of cardiomyocyte proliferation through time lapse imaging, where dividing cardiomyocytes can be visualized as they progress through the cell cycle and complete division. Methods to isolate and quantify newly formed myocytes via molecular beacons and stereological measurements, respectively, could also work collectively with these recently designed mouse models to better define true cases of cardiomyocyte proliferation.

These new approaches should be implemented in future studies to develop reliable strategies for generating new cardiac myocytes and clarifying if/when new myocytes are generated. For example, it was originally thought that cardiomyocytes retain their proliferative capacity up to 7 days after birth [8] but a study using FUCCI mice showed cardiomyocyte cell cycle activity was almost complete after postnatal day 2 with most myocytes arrested at the G1/S transition of the cell cycle [95]. In another study, it was concluded that new cardiomyocyte formation increased four-fold following MI injury [11]; reassessment of cardiomyocyte proliferation after MI in FUCCI mice showed that cardiomyocytes could indeed re-enter the cell cycle but they did not divide [95]. Therefore, these new methods should allow more precise identification of dividing myocytes and more accurate timelines of their cell cycle activity, which should aid in finding the optimal approach for inducing cardiomyocyte renewal. The ideal therapy would replenish cardiomyocyte numbers following injury, and would also promote angiogenesis, modulate inflammation, and reduce fibrosis for complete cardiac repair and regeneration.

## Figures and Tables

**Table 1:** Therapeutic stimulation of endogenous cardiomyocyte proliferation and cardiac regeneration.

Pathway	Molecular Factor	Species	Injury	Completed Division	Results	Reference
Cell cycle promoters	Cyclin D1	Mouse	No Injury	No	>40% Adult cardiomyocytes re-entered the cell cycle	[20]
	Cyclin D2	Mouse	MI	Yes	Cardiomyocyte DNA synthesis up to 5 months post-MI	[21]
	Cyclin A2	Mouse	MI	Yes, in vitro	Cardiac Hyperplasia develops in postnatal hearts	[24]
	Cyclin A2	Rat, Pig	HF, MI	Yes	~18% increase in porcine ejection fraction post MI	[25,26]
	Cyclin B1-CDC2	Rat	No Injury	No *	Increased number of small mononucleated myocytes	[27]
	CDK2	Mouse	Pressure Overload	No *	Increased number of small less-differentiated mononuclear cardiomyocytes	[28]
Cell cycle inhibitors	p21, p27, p57 (tKD)	Mouse, Rat	No Injury	Yes	Adult cardiomyocytes completed cytokinesis, expressed neonatal genes, and resembled neonatal morphology	[29]
	p21	Mouse	No Injury	No	Induced DNA synthesis without completion of mitosis	[31]
Cell cycle transcription factors	Meis1, Hoxb13 (dKO)	Mouse	MI	Yes	Increased cytokinetic and mononucleated cardiomyocytes and improved EF post MI	[37]
Combination of cell cycle regulators	4F, 2F-2i	Mouse, Rat, Human	MI	Yes	15–20% Of adult mouse myocytes re-entered the cell cycle and completed cytokinesis	[38]
Hippo	human YAP	Mouse	MI	No	Increased cardiomyocyte proliferation post MI	[39]
	hippo (KO)	Mouse	MI	Yes	Adult cardiomyocytes re-entered cell cycle	[40]
	Salv (KO, KD)	Mouse	HF	Yes *	DNA synthesis in adult myocytes up to 9 weeks post MI	[41]
Neuregulin	NRG1, ERBB4	Mouse, Rat	MI	Yes	Only mononuclear cardiomyocytes completed division	[42]
	ERBB2	Mouse	MI	Yes	Induced myocyte proliferation and redifferentiation	[43]
miRNAs	human miR-590, human miR-199a	Mouse	MI	Yes	Induced cardiomyocyte proliferation and division, and cardiac repair post MI	[44]
	human miR-199a	Pig	IR	Yes	Increased myocyte proliferation at 12 days post IR	[45]
	miR-17-92 cluster	Mouse	MI	Yes	Promoted cardiomyocyte DNA synthesis in adult mice	[46]
	miR-19a/19b	Mouse	HF, MI	Yes	Induced cardiac regeneration up to 12 months post-MI	[47]
	miR-15 family (KD)	Mouse	IR	No	Increased proliferation of adult cardiomyocytes post IR	[48]
	miR-302-367	Mouse	MI	Yes	Robust cardiomyocyte proliferation in the adult heart	[49]
	miR-301a	Mouse	MI	Yes, in vitro	Induced cell cycle re-entry of cardiomyocytes after MI	[50]
Metabolic regulators	Hypoxia	Mouse	MI	Yes	Increased cardiomyocyte division and cardiac repair	[51]
	PDK4 (KO, KD)	Mouse	MI	Yes	Enhanced cardiomyocyte proliferation post MI	[52]

miR, microRNA; KD, knockdown; tKD, triple knockdown; KO, knockout; dKO, double knockout; MI, myocardial infarction; HF, heart failure; IR, ischemia reperfusion; EF, ejection fraction; 4F represents 4 factors, i.e., CDK1, CCNB, CDK4, and CCND; 2F-2i represents 2 factors and 2 inhibitors, i.e., CDK4, CCND, MK1775 (Wee1 inhibitor), and SB431542 (TGFβ inhibitor). (*) Indicates cardiomyocyte division was indirectly implied in the reference but needs to be confirmed by experimental evidence.

**Table 2:** Strategies to measure cardiomyocyte proliferation and division.

Approach	Labeling Strategy	Advantages	Cell Cycle Phase	Limitations
Markers	EdU	Labeling cardiomyocytes that undergo DNA synthesis; EdU processing is easy, rapid, and sensitive	S	EdU also labels cardiomyocytes undergoing DNA damage and repair; no evidence of whether myocytes continue to mitosis and complete division
	Ki67	Identifies cardiomyocytes that re-entered the cell cycle	G1, S, G2	Does not identify if cardiomyocytes undergo cytokinesis
	pHH3	Labeling of cardiomyocytes that have entered Mitosis	G2, M	Unable to distinguish whether cardiac myocytes underwent cytokinesis, endoreplication, or poly-nucleation; short expression time during mitosis so low detection
	Aurora B	Present between two daughter cells during cytokinesis	G2, M, cytokinesis	Expressed during shortest phases of the cell cycle (M and cytokinesis) so low detection
	Molecular Beacons	Sorts live, isolated cardiomyocytes that are in anaphase and telophase/cytokinesis	M, cytokinesis	Unable to sort isolated cardiomyocytes from large animal models by flow cytometry
Mouse Models	MADM	Labeling of cardiomyocytes yellow (GFP + RFP) when they enter the cell cycle and upon completion of cell division, daughter cells are labeled as only green (GFP) and only red (RFP)	GFP + RFP: G1-M, only GFP and only RFP: cytokinesis	Daily or frequent tamoxifen injections can be cytotoxic for the animal
	Rainbow	Clonal expansion of labeled cardiomyocytes identifies myocytes that proliferated from pre-existing myocytes	Indirectly measures cytokinesis	Indirect way to measure cardiomyocyte division
	FUCCI	Identifies which stages of the cell cycle cardiomyocytes are present, by oscillations of an orange (mKO) and green (AzG) color within cardiomyocyte nuclei	mKO: G1, AzG: S, G2, M; mKO + AzG: G1/S cell cycle arrest	AzG labeling unable to distinguish whether myocytes are in S phase, G2 phase, or M phase; inability to visualize cytokinesis
	Aurkb	Cardiomyocytes are labeled red when they undergo proliferation and division; Cytokinesis visualized by live time lapse imaging	S, G2, M, cytokinesis	Inability to distinguish whether myocytes in vivo are in S phase, G2 phase, M phase, or cytokinesis; completed cytokinesis limited to live cell imaging
3D Imaging	Stereology	Estimates the number of cardiomyocytes in the heart using representative thick sections	Indirectly measures new myocyte numbers	Accuracy of cardiomyocyte number is limited

Phases of the cell cycle consist of G1 (cell growth), S (DNA synthesis), G2 (cell growth and preparation for mitosis), and the mitotic phase that includes M (mitosis, nuclear division) and cytokinesis (cytoplasmic division). EdU, 5-ethynyl-2′-deoxyuridine; pHH3, phosphorylated histone H3; MADM, mosaic analysis with double markers; FUCCI, fluorescence ubiquitin cell cycle indicator; Aurkb, Aurora kinase B; GFP; green fluorescent protein; RFP, red fluorescent protein; mKO, monomeric Kusabira Orange; AzG, Azami Green.
